# Lower back pain prevalence and experiences in civil service workers, Botswana

**DOI:** 10.4102/phcfm.v17i1.4629

**Published:** 2025-04-17

**Authors:** David Damba, Sonti I. Pilusa

**Affiliations:** 1 Department of Physiotherapy, Faculty of Health Sciences, University of the Witwatersrand, Parktown, South Africa

**Keywords:** lower back pain, workers, well-being, work performance, civil service

## Abstract

**Background:**

Lower back pain (LBP) is a global problem contributing to both disability and an increased demand for rehabilitation services. Studies on the prevalence and impact on work performance in Botswana are scarce.

**Aim:**

We investigated the prevalence of LBP among civil workers in Botswana and their experiences.

**Setting:**

The study setting comprised physiotherapy practice, Gaborone, Botswana.

**Methods:**

Concurrent explanatory mixed methods were employed. A total of 339 medical files for civil service workers were reviewed to identify the prevalence of LBP and associated risk factors such as age, gender, body mass index, working duration and type of work. Descriptive statistics were performed. In addition, 20 civil service workers were interviewed. The interviews were transcribed verbatim and coded inductively.

**Results:**

A total of 339 files were reviewed and the prevalence of LBP was 49%. There was no association between the risk factors and LBP (*p* > 0.05). The experience of LBP was expressed in terms of the characteristics of LBP; the effects of LBP on all life domains and how LBP was managed. Our findings were that LBP affected work life and work performance.

**Conclusion:**

The high prevalence of LBP in civil service workers is concerning because it affects all the domains of life including work performance. Workplace health and wellness interventions are recommended.

**Contribution:**

Workplace health and wellness interventions are recommended.

## Introduction

Globally lower back pain (LBP) is the most common musculoskeletal condition and a leading public health burden.^1^ Lower back pain involves acute or chronic pain or discomfort in the lower lumber region of the back. In 2017, 577 million people globally experienced LBP with a point prevalence of 7.5%.^1^ In Africa, the prevalence of LBP was estimated to be 39%, higher than global trends.^2^ Greater awareness of LBP is needed as it is postulated that there will be an increased prevalence of LBP in the future because of an increase in the ageing population, urbanisation and chronic health conditions associated with LBP.

Lower back pain prevalence increases with age among the working population resulting in increased disability.^1,3^ African-based studies looking at the prevalence of LBP among workers reported high prevalence.^4,5^ In Kenya, the prevalence of LBP was reported to be 64.9%.^4^ In Ethiopia, LBP among civil service workers was 39%.^5^ In Botswana, the reported prevalence of LBP was 56% among teachers and 69% among cashiers, respectively.^6,7^ Studies looking at other cadres of civil service workers have not been conducted in Botswana. Having research that highlights the prevalence of LBP among other cadres, such as administrators, can inform civil service workplace interventions to minimise the negative impact of LBP on work life.

There is evidence showing that LBP affects different life domains.^8^ The International Classification of Functioning and Disability and Health presents aspects of life that can be affected by health conditions such as activities of daily living, mobility, carrying a bucket of water, standing for long and the individual’s role in life, for example, going to work, attending church or social events. Activities such as sleeping, cleaning and gardening may also be negatively impacted as LBP restricts movement.^7^

The impact of LBP can result in reduced productivity among the working population. The presence of LBP can affect the workers’ ability to carry out work-related activities thus affecting workers’ efficiency, performance and absenteeism levels and increasing the risk for early retirement.^3,8^ Given the interconnectedness of life domains, personal finances are lost through seeking healthcare. Workers spend most of their time at work, and understanding the impact of LBP on worker wellness is important for the development and improvement of worker wellness programmes.

Disability because of LBP is projected to increase, consequently increasing the demand for rehabilitation care.^9^ Most of the time LBP is managed medically through pharmaceutical approaches.^10^ However, people living with LBP can benefit from a person-centred, bio-psychosocial approach to care where the determinants of LBP such as age, poor posture, obesity, non-communicable diseases, smoking and poor ergonomics can be addressed.^3^ In addition, a long-term perspective on LBP management through promoting self-management strategies and rehabilitation care can play an important role in minimising the associated risk factors and disability. A local understanding of the prevalence of LBP and its effects is important to develop context-based interventions. Thus, the objective of this study was to establish the prevalence of LBP and explore the impact of LBP on the well-being of civil service workers in Botswana.

## Research methods and design

### Study design

This study used a concurrent explanatory mixed-method approach incorporating a record review and qualitative design using semi-structured interviews to better understand LBP and its impact.^11^

### Study setting

The study was conducted at a private physiotherapy practice based in Gaborone Botswana. The practice offers physiotherapy services to address neurological conditions and musculoskeletal problems to those who have either medical insurance or who can afford these services privately. Some of the service users include government civil service workers.

### Study population

Medical records of civil service workers at a private physiotherapy practice based in Botswana were reviewed to determine the prevalence of LBP and the associated risk factors. We included medical records of civil service workers from 01 January 2018 to 30 January 2021. In addition, civil service workers aged 18–60 years with LBP who used the private physiotherapy practice were purposively invited to participate in online semi-structured interviews.

### Data-collection tools

For the record review, a data extraction sheet was designed by the researchers based on previous studies on LBP and validated for content and face validity by two senior physiotherapists with experience in research and over 10 years in physiotherapy practices. The questions in the data extraction sheet included the demographic profile; LBP (date of onset, severity, pain intensity, treatment modality, presence of pain, intensity and location of pain) and presence of other chronic health conditions was used to collect data from the medical files.

For the semi-structured interviews, an interview guide with open-ended questions on LBP; LBP management and the influence of LBP on work and work performance was used to facilitate the semi-structured interviews.

### Interview guide questions with probes

*Tell me about yourself and your work. Probes: what does your work entail/how long have you been working at your workplace?*

*Please tell me about the LBP that you are experiencing. Probes: Elaborate on when and how LBP started. (Details on the LBP triggers, severity, location)/How often do you experience low back pain? /What makes the LBP worse?*

*Please tell me how you manage the LBP. Probes: can you share with me how you make LBP better?/How does LBP affect your life in general?*

*Please can you share with me how LBP affects your work life? Probes: Work routine?/Does LBP affect your job performance?/How does LBP affect your ability to do your work?*

### Data collection

The data-extraction tool was piloted before the main study by the principal researcher. The files that fit the inclusion criteria were identified and permission to review the files was sought from the respective patients. Relevant data were extracted using the data-extraction sheet. A test–retest reliability method was employed and conducted over some time on the same data set.

The participants with LBP whose files were included in the record review were contacted telephonically by the principal researcher, explaining the study aim and were invited to participate in the telephonic or face-to-face semi-structured interviews. Notes were taken during the interviews and the researchers held regular debriefing sessions throughout the research process. All the interviews were audio-recorded and lasted approximately 20 min, adhering to coronavirus disease 2019 (COVID-19) precautions. Interviews were conducted from January 2022 to February 2022. Trustworthiness was ensured by audio recording all the interviews; regular briefing meetings between the researchers and an in-depth description of the data-collection process.

### Data analysis

Statistical Package for Social Services (SPSS) version 22 was used for descriptive analyses, significance was set at *p*-value < 0.05 and data were presented in frequency, percentage, median and interquartile range (IQR). Chi-squared test and Fischer’s exact test were conducted to assess the associations between LBP and the associated risk factors. All the audio-recorded interviews were transcribed verbatim and exported to Max Weber Qualitative Data Analysis (MAXQDA) version 2020 software for analyses. We analysed the transcripts separately and compared the findings. The transcripts were coded inductively by identifying meaning units, condensing them and identifying categories.

### Ethical consideration

Ethical clearance was granted by the Human Research Ethics Committee (Medical) of the University of the Witwatersrand (number M210910); the Ministry of Health of the Republic of Botswana and the private physiotherapy practice administrator granted permission to conduct the research before data collection. The researchers explained the study to the potential participants and asked for consent to review their files and those who met the criteria for semi-interviews were asked to participate in the interviews. Verbal consent was given for the interviews and recording of the interview. Participation in the study was entirely voluntary and participants were free to decide to take part in the study and were also free to withdraw from the study at any time and nothing was to be held against them. To ensure confidentiality and non-disclosure of data from the study to third parties, the researchers used pseudonyms instead of actual names for all the participants

## Results

We reviewed 339 medical files comprising 69% females and 31% males. Median age (IQR) was 46 (39–52) years. Forty-nine per cent (165 participants) of the workers had LBP. There was no association between the participants’ demographic profile, body mass index (BMI), medical co-morbidities and LBP (*p* > 0.05). Table 1 illustrates the workers’ demographic profile.

**TABLE 1:** Civil service workers’ demographic profile.

Demographic profile	Median	IQR	*n*	%
**Age *n* (%)**				
Median (IQR)	46	39–52 years	-	-
≤ 35 years	-	-	49	14.5
36–45 years	-	-	119	35.1
46–55 years	-	-	134	39.5
≥ 55 years	-	-	37	10.9
**Gender**				
Female	-	-	234	69.0
Male	-	-	105	31.0
**Marital status**	-	-	-	-
Single	-	-	150	44.2
Married	-	-	185	54.6
Widowed	-	-	4	1.2
**Occupation**				
Office jobs	-	-	139	-
Teachers	-	-	91	-
Security	-	-	44	-
Health workers	-	-	33	-
Manual labourer	-	-	19	-
IT/Engineering	-	-	13	-
**Body mass index**				
Normal	-	-	193	56.9
Overweight	-	-	91	26.8
Obese	-	-	55	16.2
**Presence of co-morbidities**				
Diabetes	-	-	17	5.0
HIV	-	-	2	0.6
Hypertension	-	-	103	30.4
Lower back pain	-	-	165	48.6

IQR, Interquartile range; IT, Information technology; HIV, human immunodeficiency virus.

In part 2, the qualitative study, 20 civil service workers with LBP were interviewed. The mean age was 40.5 years with standard deviation 10.87. There were ten males and ten female participants, while 50% of the participants were married. Table 2 illustrates the participants’ profile.

**TABLE 2:** Interviewed participants’ work profile.

Demographic profile *n* (%)	Mean	Range	s.d.	*n*	%
**Age (years)**					
Mean	40.5	-	-	-	-
Range	-	35	-	-	-
s.d.	-	-	10.87	-	-
**Gender**	-	-	-	-	-
Male	-	-	-	10	50
Females	-	-	-	10	50
**Marital status**					
Single	-	-	-	10	50
Married	-	-	-	10	50
**Work type of the participants**					
Executives (E)	-	-	-	4	20
Skilled workers (SW)	-	-	-	9	45
Semi-skilled workers	-	-	-	5	25
Support staff	-	-	-	2	10
**Working hours duration**					
Mean	7.7	-	-	-	-
Range	-	4	-	-	-
s.d.	-	-	1.33	-	-
**Duration of work years**					
Mean	13.35	-	-	-	-
Range	-	43	-	-	-
s.d.	-	-	11.65	-	-
**Duration of work years**					
1–10	-	-	-	12	60
11–20	-	-	-	4	20
21–30	-	-	-	1	5
> 31	-	-	-	3	15

s.d., standard deviation.

The experience of LBP was expressed in three categories: characteristics of LBP, the effects on all life domains such as sleep, mental health, mobility, social life and finances and how LBP was managed.

### Lower back pain characteristics

The participants described how they experienced LBP as an intense, shooting, burning and cramping pain: ‘The lower back pain is severe and it even shoots to the thighs’ (Peyton, 44 years old, civil service) and ‘if the cramps come up, a lot of pain flashes down along my right leg as if it was a nerve pulse’ (Percy, 32 years old, civil service).

For some of the participants’ LBP was constant and for some it was intermittent:

‘So, it comes, and it goes, and I always come to the Physio to massage when I have that severe pain.’ (Polk, 60 years old, civil service)

‘I have had lower back pain for a long time. The pain is off and on. I have learnt to live with it.’ (Pablo, 34 years old, civil service)

#### Causes of lower back pain

The participants stated the causes of LBP to include prolonged postures, surgery, injury, incorrect lifting techniques and work-related stress. For example, Mercia said:

‘Lower back pain started after the delivery of my baby, I was given anaesthesia injection on my lower back spine for C-section early 2019 and since then I suffered from the lower back pain but was taking some pain killers.’ (Mercia, 36 years old, civil service)

Martha’s LBP was caused by a past injury from a fall and lifting a heavy object ‘because of the fall and that is why I am experiencing a lot of pain, and the pain never subsides (Martha, 60 years old, civil service). Most of the participants reported that prolonged sitting and standing positions, bending and stressful work situations worsened the LBPs, ‘It becomes worse during the month end where I have to sit long hours compiling some reports’ (Mary, 44 years old, civil service).

### The effects of lower back pain on all life domains

**FIGURE 1:**
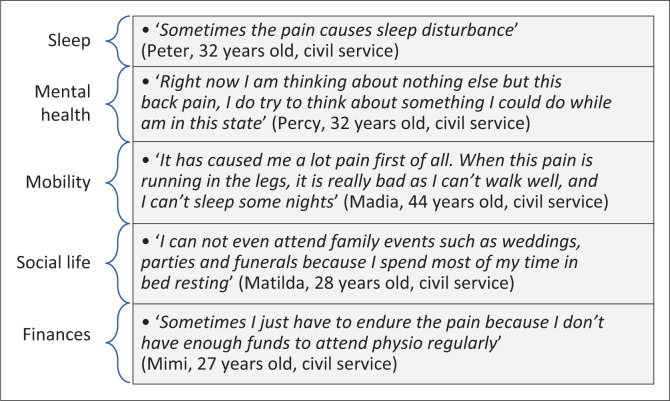
Quotes for the effects of lower back pain on all domains of life.

The workers highlighted how LBP affected all life domains – sleep, mental health, mobility, social life and their finances and work life (see Figure 1).

### Lower back pain and work-life

All the participants highlighted how LBP negatively affected their work life. Lower back pain affected the workers’ ability to perform some of the work-related activities:

‘Well, my work requires standing, and I cannot do much standing and a lot of patrolling and my steps as I walk are not meeting the requirements for am taking small steps.’ (Percy, 32 years old, civil service)

Lower back pain decreased the pace some of the workers could work and decreased their work output ‘I can’t work at the rate I used to work …, so that means my financial capacity reduces because of that’ (Patrick, 51 years old, civil service).

Some participants highlighted that when the pain was severe, they could not come to work:

‘I can’t do my normal duties when the back is hurting, I definitely can’t perform properly when I have back pain, if it is severe, I have to get sick leave.’ (Mercy, 36 years old, civil service)

Experiences of LBP increased job insecurity ‘LBP is bothering me because it is putting my job on the line’ (Paco, 36 years old, civil service) and other participants considered early retirement ‘Aaaa honestly the back pain has affected my opportunities to be promoted … I will have to go for early retirement, it is allowed in our service’ (Madia, 44 years old, civil service).

### Lower back pain management

**FIGURE 2:**
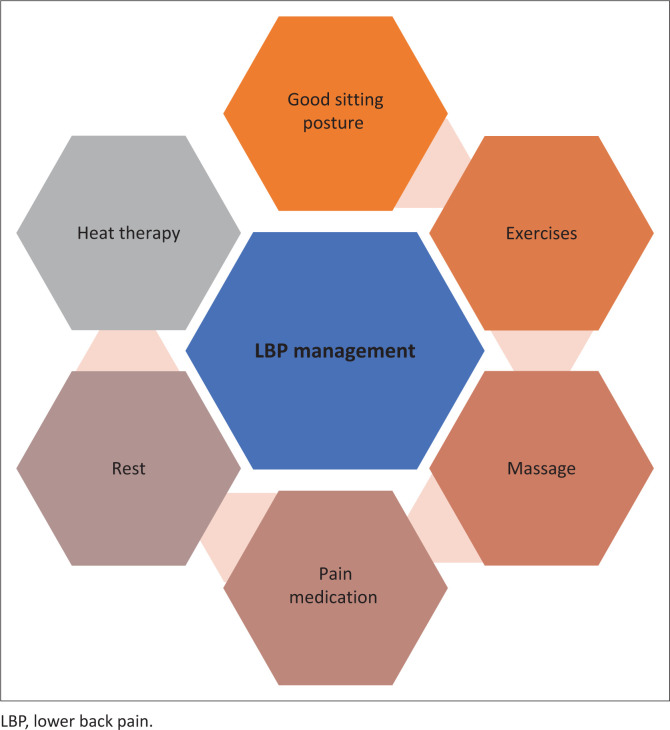
Lower back pain management strategies.

Besides attending physiotherapy and seeing a medical doctor, the participants used multiple strategies to manage LBP (see Figure 2). However, some participants stated that they had to learn to live with LBP, ‘I have had lower back pain for a long time. The pain is off and on. I have learnt to live with it’ (Pablo, 34 years old, civil service). Maintaining a good sitting posture, doing exercises, massage, pain medication, rest, change of posture or positioning, heat therapy, massage, exercises and attending physiotherapy helped in relieving LBP.

## Discussion

To the authors’ knowledge, this is the only study in Botswana looking at LBP in civil service workers. This study aimed to establish the prevalence of LBP in civil service workers and explore its effects on work performance. Forty-nine per cent of the civil service workers had LBP. This percentage is slightly higher than the lifetime and point prevalence of LBP in Africa (47% and 39%, respectively).^2^ Compared to other studies based in Botswana, the prevalence of LBP among civil workers was lower than the reported prevalence among teachers and cashiers.^6,7^ The difference in the results could be because of methodological differences, we used a retrospective study and the data collected from the medical records were from civil service workers who had access to the private practice. The prevalence is high indicating a need for workplace prevention strategies to minimise the occurrence and address LBP-related disability. Future prevalence studies using stronger methodologies like longitudinal studies and a larger study population are recommended to ascertain the extent of LBP in the civil service worker population.

Firstly, contrary to previous studies that reported associations between age, gender and education we did not find any associations between demographic profile and LBP.^12,13,14^ Secondly, there was no association between LBP and the presence of co-morbidities (BMI, HIV, Diabetes and Hypertension). The findings contradict previous reports on the associations between LBP and co-morbidities.^15^ Lower back pain has been reported to be associated with BMI, hypertension, diabetes and HIV.^5,15,16^ The difference may be because the methodology employed in this study was based on a smaller population in a private practice and over a short period. Future studies are recommended to explore LBP among workers in Botswana and to include other risk factors such as psychological and occupational risk factors such as stress and repetitive motions. Studies of this nature will inform wellness programmes for workers.

Like previous studies, we found that LBP affected all domains of life namely physical, social, financial, mental health and work life.^4,17^ People with LBP experience serious functional limitations and restrictions in the participation domains including work disability.^8^ The economic cost of LBP should not be underestimated for the individual, the family, and the health care system. The devastating impact of LBP points to the need for a systems approach to LBP prevention and care. This was confirmed by the call to action to address the LBP burden through various strategies such as prioritising the prevention of LBP, developing interventions to address risk factors; changing workplace practice and reorienting the health systems to promote person-centred care.^18^

Management of LBP tends to be mostly through medication because of the lack of awareness of the role of rehabilitation in the health system and poor access to rehabilitation care. It was good to see that the participants in our study used various strategies to manage LBP besides medication. They used good posture, exercising, massage, rest, positioning, heat therapy and attending physiotherapy. Taking into consideration the fact that LBP can be a chronic disabling condition, a bio-psychosocial approach is important.^19^ Rehabilitation interventions should include self-management, healthy living practices (increasing physical activity, smoking cessation and weight loss), psychological support, health education on pain management and correct ergonomics.^19^ Work-based interventions such as workplace wellness programmes; regular risk assessments; ergonomic assessments and ergonomic education to minimise the occurrence of LBP are recommended. Future research in LBP can assess environmental risk factors and assist in designing safe workplaces.

### Strengths

The strength of this study lies in the fact that it is the first study to use a mixed-method design to quantify LBP and explore experiences related to LBP in civil service workers in Botswana.

### Limitations

The study was based in one setting, a private practice, therefore it excluded civil service workers who use the public health system. The qualitative findings are the experiences of the study participants and cannot be generalised. We used paper-based records for the record review and as a result important information related to LBP and risk factors might not have been recorded. The use of telephone in both data collections also posed challenges such as interview questions being short, which yields brief responses from the respondents.
